# Capsaicin-induced CGRP-mediated vasodilatation of the human skin: influence of gender, female hormones and migraine

**DOI:** 10.1186/1129-2377-14-S1-P124

**Published:** 2013-02-21

**Authors:** SGG Vermeersch, P Frederiks, A Maassen VandenBrink, J de Hoon

**Affiliations:** 1 Center for Clinical Pharmacology, University Hospitals Leuven, Campus Gasthuisberg, (KU Leuven), Belgium; 2 Division of Vascular Medicine and Pharmacology, Department of Internal Medicine, Erasmus MC, Netherlands

## 

Migraine is much more common in women than in men. It is associated with changes in female sex hormone levels with peaks of migraine frequency occurring when estrogen levels drop. Calcitonin gene-related peptide (CGRP) is a potent vasodilating neuropeptide with a pivotal role in migraine headache. Endogenous release of CGRP is induced by capsaicin through activation of the transient receptor potential vanilloid 1 (TRPV1) channel. This study aimed to investigate the influence of gender, hormonal changes and migraine on dermal blood flow (DBF) resulting from capsaicin-induced release of CGRP.

Healthy, non-smoking female volunteers (n=16) not using hormonal contraceptives, were investigated weekly during 2 menstrual cycles. Weekly, two doses of capsaicin (300 and 1000µg) and vehicle were applied topically on the skin of both forearms. DBF was assessed before and at 10, 20, 30 and 40 minutes after capsaicin/vehicle application using laser Doppler imaging. DBF is expressed as percentage increase versus baseline and presented as area-under-the-curve from 0 to 40 minutes (AUC, %.min, mean±SEM).

Period differences in capsaicin-induced DBF were observed after both doses of capsaicin (p<0.001, repeated-measures ANOVA). During menstruation, capsaicin-induced DBF, expressed as AUC, was larger after 300µg (1488±178 versus 1228±157 %.min, p=0.019; paired T-test) and 1000µg of capsaicin (1639±150 versus 1394±163 %.min, p=0.014) compared to the second week of the secretory phase of the menstrual cycle. Analyses of gender differences and migraine patients versus healthy subjects are ongoing and will be presented at the meeting.

## Conclusion

In healthy women, a hormonal influence on capsaicin-induced CGRP-mediated vasodilation of the skin is observed. In particular, an increased dermal blood flow response is documented during the menstruation period. This could be the result of increased neuronal sensitivity to capsaicin, increased release of CGRP or increased sensitivity to CGRP. These results support the hypothesis that female hormones are related to the susceptibility to migraine.
